# Thermal Sight: A Position‐Sensitive Detector for a Pinpoint Heat Spot

**DOI:** 10.1002/smsc.202400091

**Published:** 2024-07-11

**Authors:** Jun Peng, Pai Zhao, Rakshith Venugopal, Kristian Deneke, Stefanie Haugg, Robert Blick, Robert Zierold

**Affiliations:** ^1^ Center for Hybrid Nanostructures Universität Hamburg 22761 Hamburg Germany; ^2^ Institute for Materials and X‐ray Physics Hamburg University of Technology 21073 Hamburg Germany; ^3^ DESY Photon Science Deutsches Elektronen‐Synchrotron DESY 22607 Hamburg Germany

**Keywords:** atomic layer deposition, heat spot, heat transfer, position‐sensitive detector, thermoelectric

## Abstract

Precise positioning is a never‐ending goal in both fundamental science and technology. Recent decades of advancements in high‐precision position detection have predominantly relied on photoelectric effects for light detection in semiconductors. Herein, a different approach is proposed: The thermoelectric‐based position‐sensitive detector (T‐PSD) concept is designed to detect single heat spots arising from various energy sources, including electromagnetic radiation, electrons, and macroscopic mechanical heat. The T‐PSD concept is initially derived mathematically from the fundamental principles of heat conduction and the Seebeck effect. Subsequently, it is proved by finite element simulation in both 1D and 2D configurations. Following this theoretical groundwork, T‐PSD prototypes are fabricated and subjected to positional detection using various stimuli such as CO_2_ laser beam, hot soldering tip, and electron beam. In the prototypes, structured aluminum‐doped zinc oxide thermoelectric thin films, prepared via atomic layer deposition, are outfitted with voltage probes, enabling the measurement of thermoelectric voltages as a function of position and the intensity or temperature of the heat spot. Furthermore, practical decoding strategies are introduced to infer the position from the measured signals. The T‐PSD in this article showcases considerable promise in high‐precision position detection such as (quasi‐)particle tracking and precision machinery, offering an alternative concept in PSD design.

## Graphical abstract

This study proposes a thermoelectric‐based position‐sensitive detector concept, providing an alternative but more versatile approach to high‐precision position detection compared to existing detectors. Utilizing structured aluminum‐doped zinc oxide thermoelectric thin film prepared via atomic layer deposition as an amplifier, the prototypes exhibit accurate detection of single heat spots originating from electromagnetic radiation, electrons, and macroscopic mechanical heat sources.

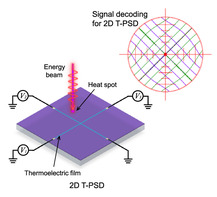

## 1 Introduction

**Figure 1:**
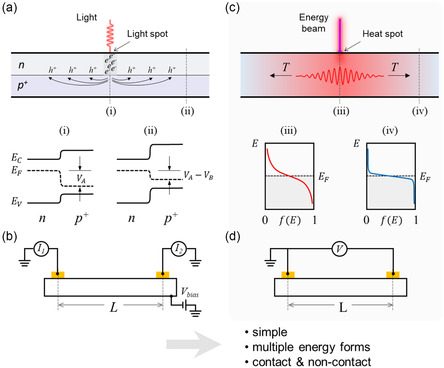
Comparison of conventional PSD and our T‐PSD. a) The working principle of conventional PSD based on the lateral photoelectric effect, exemplified by the junction between an n‐type region (*n*) and another more heavily doped *p*‐type region (*p+*). When a beam of light illuminates the junction, hole‐electron pairs are generated, establishing a new steady‐state condition where most of the injected holes accumulate in the *p+* region and most of the injected electrons reside in the *n* region. This results in a shift in the Fermi levels (i) at the light spot and (ii) away from the light spot, leading to a transverse photovoltage *V*_A_‐*V*_B_ between the two positions. b) A simplified photocurrent circuit for light spot position estimation. c) The working principle of our T‐PSD. In the presence of a HS on the detector surface, phonons are continuously excited and propagate through the detector, making that the Fermi distribution (iii) at the HS is “softer” than that (iv) at the cooler surrounding area. This non‐equilibrium distribution of hot electrons and holes results in thermoelectric voltage which is solely dependent on the temperature difference between two points within the detector. Importantly, the HS can be generated by various forms of energy. d) The simplified circuit to detect the thermoelectric voltage for HS position estimation.

Position‐sensitive detectors (PSDs) are devices that can determine the position of incident radiation or (quasi‐)particles in a spatially sensitive manner. These devices serve as fundamental components in modern industry and science. They hold a significant prominence throughout numerous applications, such as motion tracking,^[^ ^1^ ^]^ 3D printing,^[^ ^2^ ^]^ robotic,^[^ ^3^ ^]^ machining,^[^ ^4^ ^]^ and (quasi‐)particle detection.^[^ ^5^ ^]^ State‐of‐the‐art PSDs are typically based on the lateral photoelectric effect in a semiconductor junction (**Figure** **1**a,b).^[^ ^6^ ^]^ Following the existing main PSD design principles, they can be primarily classified into two groups. The first category involves the lateral photoelectric effect on an isotropic sensor surface, supplying continuous position data. Typical representatives of this design are lateral PSD^[^ ^7^ ^]^ and quadrant PSD.^[^ ^8^ ^]^ They detect the light spot position by estimating the photocurrents measured by several electrodes. Such PSDs are widely used in ranging systems or high‐precision instruments,^[^ ^9^ ^]^ etc. The second principle is to integrate discrete detection units together as a PSD to extract position information via pixel imaging, albeit at an expensive cost and inevitably sacrificing resolution. In the consumer market, for instance, complementary metal‐oxide‐semiconductors (CMOS) chips^[1b]^ incorporate millions of small discrete pixels, so‐called PIN diodes, as detection units to determine the light position. However, these detectors, relying on silicon‐based materials, impose strict requirements, i.e., limited operating temperature range and wavelength detection range.^[^ ^10^ ^]^ Other more complex discrete PSDs, for example, thermopile arrays^[^ ^11^ ^]^ and bolometer arrays,^[^ ^12^ ^]^ can work in wider operating temperatures and spectral range, but still suffer from the complex fabrication process and low resolution.

This work introduces an innovative concept for a PSD derived from the heat transfer principles, which detects the position of a single heat spot (HS) on the detection surface precisely. Isotropic heat conduction on a homogeneous substrate results in a uniform temperature gradient distribution around a HS. Consequently, the HS position on the surface of the substrate can be determined based on the thermoelectric voltages generated from the temperature differences between HS and pre‐defined electrodes (Figure 1c,d). Based on this idea, we developed a new type of PSD, realized in 1D and 2D devices, for single‐point HS detection, termed the thermoelectric‐based PSD (T‐PSD). Since the arising thermoelectric voltages determine the signal strength, a thermoelectric thin film is incorporated into the detector design to amplify the measured signal. The herein proposed mathematical model for T‐PSD is validated through both finite element analysis (FEA) and experiments on the 1D and 2D T‐PSD prototypes. Furthermore, an approach for decoding the measured voltage signals to obtain a specific position is demonstrated. As the T‐PSD relies on temperature differences for detection, this work demonstrates its capabilities in detecting HSs converted from various energy forms.

## 2 Results and Discussion

### 2.1 Design Principle for T‐PSD

The T‐PSDs developed herein rely on the basic principles of heat conduction and the Seebeck effect, enabling accurate determination of a HS on the detector surface. Assuming an ideal situation where only heat conduction is considered, a HS is introduced to an isotropic plate surface. On the one hand, energy is transferred from hotter regions near the HS to those farther away and at lower temperatures. The temperature distribution on the plate is described by Fourier's law of heat conduction^[^ ^13^ ^]^

(1) $$q_{x}=-kA\frac{dT}{dx}$$

where *q*_*x*_ represents the rate of heat transfer, *k* denotes the thermal conductivity, *A* corresponds to the cross‐sectional area through which the heat flows, and $\frac{dT}{dx}$ indicates the temperature gradient in the direction of the heat flow. On the other hand, a thermoelectric voltage arises when a temperature difference exists across two points within the plate, a phenomenon known as the Seebeck effect,^[^ ^14^ ^]^ expressed as

(2) $$S=-\left(\frac{dV}{dT}\right)$$

In the above equation, *S* is the Seebeck coefficient, while *dV* represents the thermoelectric voltage arising from the temperature difference *dT* between the two points. The relationship between *dV* and *dx* can be quantitatively concluded from Equation (1) and Equation (2) if *k* and *S* are independent of temperature. The resulting relation builds the core principle of our T‐PSDs, allowing precise derivation of the HS position on the detector surface. By appropriately extending Fourier's law, mathematical models for 1D and 2D T‐PSDs can be derived.

The realization of T‐PSD requires a meticulous selection of the fabrication materials involved. For the substrate material, in addition to being thermally isotropic, it also needs a significant *S* to make the detected thermoelectric signal more sensitive to a temperature change. Therefore, we developed a strategy for using an isotropic substrate with a thermoelectric thin film on top. In this way, *k* and *S* are mainly determined by the substrate and the thermoelectric thin film, respectively. Here, a silicon wafer with a 300 nm oxide insulating layer is used as the substrate, and an alumina‐doped zinc oxide (AZO) film,^[^ ^15^ ^]^ with stable chemical properties in air, is selected to be the thermoelectric film. To minimize the potential contact thermal resistance^[^ ^16^ ^]^ between the substrate and the film, atomic layer deposition (ALD), with its inherent conformal coating characteristic^[^ ^17^ ^]^ is chosen to deposit the thermoelectric film. Following that, we systematically scrutinize these models using a combination of FEA simulations and actual prototypes. For theoretical simplicity, the theoretical model considers only the heat conduction within the substrate, neglecting impact from external, environmental factors on the test signal. Hence, our approach does not account for fluctuations in the ambient temperature or electromagnetic fields affecting the substrate's temperature and obtained electrical potential distribution, respectively. However, subsequent experimental validation proves the model's reliability and accuracy under stable temperature and electromagnetic field conditions.

### 2.2 Implementation and Multiple Energy Form Verification for 1D T‐PSD

**Figure 2:**
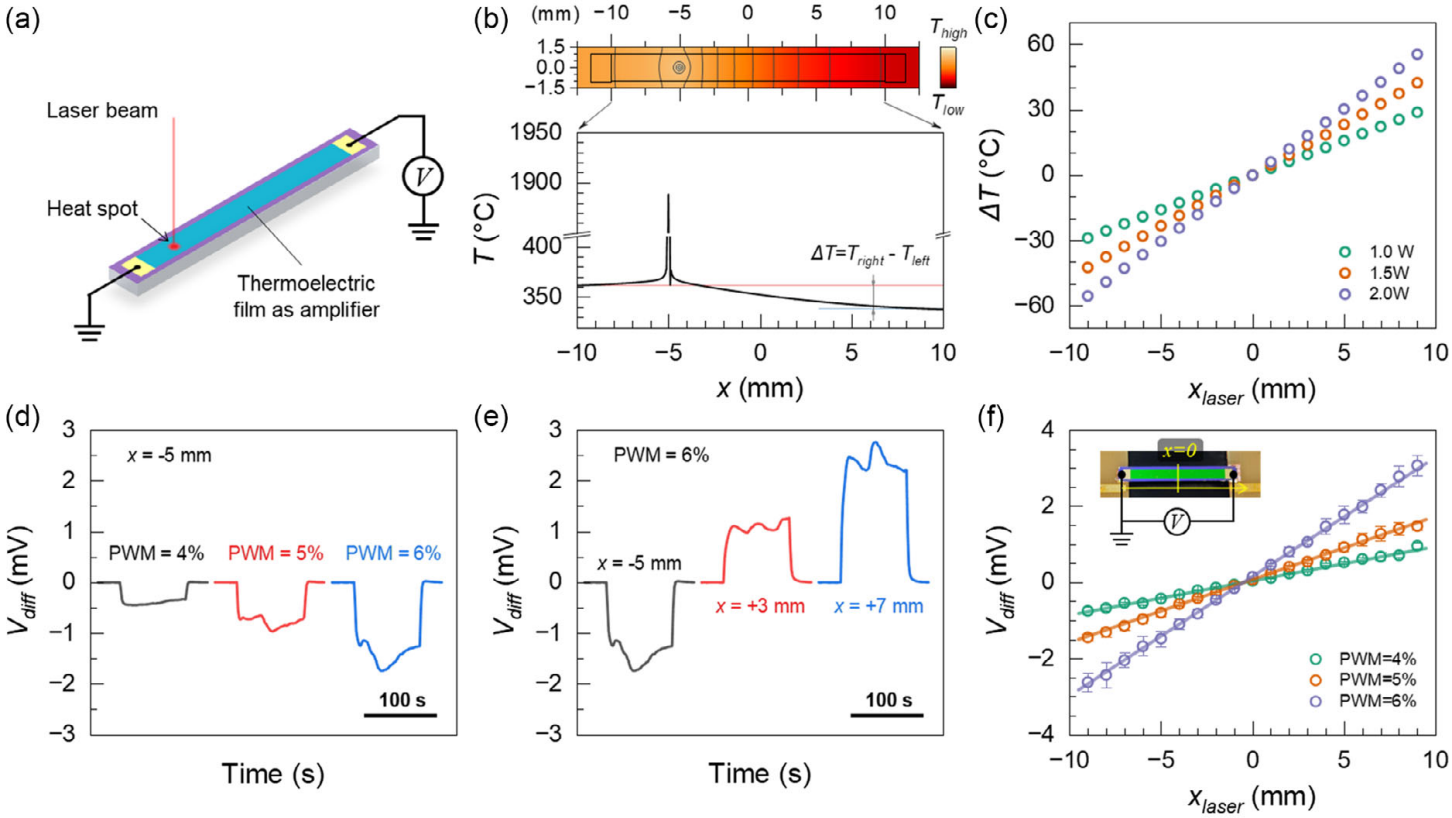
1D T‐PSD simulation and experimental results. a) Working principal sketch of a 1D T‐PSD using a laser as a HS. b) The simulated temperature distribution on the 1D T‐PSD with a HS at *x* = −5 mm and its distribution along the detector. A temperature difference, $\Delta T=T_{\text{right}}-T_{\text{left}}$, between the left terminal and the right terminal exists upon irradiating with a HS. c) Simulated Δ*T* as a function of the HS position on the 1D T‐PSD surface. d) Experimentally derived *V*_diff_ when the laser spot is located at *x* = −5 mm for different PWM values of 4%, 5%, and 6% of a CO_2_ laser. e) Measured *V*_diff_ at a laser PWM = 6% at different positions, *x* = −5,+3 and +7 mm. f) Measured *V*_diff_ using different PWM values along the detector. The lines are linear fits with *R* ^2^ ≈ 0.94, 0.92, and 0.88. Inset shows the photograph of the tested 1D T‐PSD prototype.

The 1D T‐PSD design, depicted in **Figure** **2**a, consists of a homogeneous substrate, a thermoelectric film on top, and two electrodes positioned at each end. According to the theoretical derivation outlined in Note 1.1, a HS located at *x* with a constant temperature *T*_0_ leads to a thermoelectric voltage $V_{\text{diff}}=V_{\text{right}}-V_{\text{left}}$ between the two terminal electrodes, which can be expressed as

(3) $$V_{\text{diff}}=\frac{qS}{kwd}\times 2x$$

where *q* is the heat flux density and *S* denotes the Seebeck coefficient of the thermoelectric thin film. *k*, *w*, and *d* correspond to the thermal conductivity, width, and thickness of the substrate, respectively. *x* is the position of the HS. To provide more evidence and clarify our concept, an FEA model is constructed (Figure S1.1, Supporting Information). Upon introducing a HS, for example, a laser beam, i.e.,  typically applied in laser cutting, to the sensor area, the temperature distribution becomes uneven, resulting in a temperature difference, $\Delta T=T_{\text{right}}-T_{\text{left}}$, between the two terminals (Figure 2b). The change in this Δ*T* is a power‐dependent, linear function of the position as displayed in Figure 2c. This change also results in a linear relationship between the corresponding measurable *V*_diff_ and the HS position (Figure S1.2, Supporting Information). *V*_diff_ is zero when the HS is at the center of the 1D T‐PSD, i.e., *x* = 0. When the HS deviates in one direction from the center, it yields a signal of one polarity, while a signal of the opposite polarity is produced when the HS is displaced in the opposite direction. As a result, the 1D sensor is capable of precisely detecting the center position of a HS by probing the null signal.

To support our analytical and simulation results, we fabricated 1D T‐PSDs and evaluated them using a custom‐built setup (Figure S1.3, Supporting Information). Note, all characterizations were performed in an electromagnetic‐shielded, clean, and dry environment with a controlled, stable ambient temperature. The measured *V*_diff_ reveals a sensitive response to a CO_2_ laser spot serving as a HS on the sensing surface. The sensitivity is evident in the signal derived from the laser's different pulse width modulation (PWM) values (Figure 2d) and across various positions (Figure 2e). Note that a smaller PWM in the CO_2_ laser system corresponds to a lower laser power output, thus leading to lower actual temperatures on the T‐PSD. The detected signal exhibits minor fluctuations during the laser's ON state owing to the intermittent nature of the laser (Figure S1.4, Supporting Information). The ability to detect these fluctuations showcase our sensor's exceptional sensitivity. The measured *V*_diff_ exhibits a linear relationship with the position on the T‐PSD, as shown in Figure 2f. The minor deviations in the central position at *V*_diff_ = 0 are primarily attributed to the influence of the temperature distribution of the imperfect geometry of the substrate. The most noteworthy finding is the high reproducibility of these measurements for different PWM values, namely, 4%, 5%, and 6%, with corresponding central offsets of *x* = (−0.46 ± 0.03), (−0.47 ± 0.02) and (−0.50 ± 0.01) mm, respectively. These results indicate that the central position remains constant irrespective of the absolute temperature or fluctuation of the HS. This consistency aligns with the predictions made by the theoretical models. The position sensitivity of the T‐PSD is quantified by the slope of the line, expressed in units of V mm^−1^

(4) $$\frac{dV_{\text{diff}}}{dx}=-\frac{2qS}{kwd}$$

Our 1D T‐PSD has a positional sensitivity of 0.313 mV mm^−1^ at a PMW value of 6%. Based on the multimeter's resolution of 100 nV and the specific parameters of the materials used, the detection resolution for the HS's central position is estimated to be 0.319 μm. Note, the resolution may be further enhanced by employing a thermoelectric film with a higher *S*, a substrate with lower *k*, a thinner *d*, and a narrower *w*, as indicated by Equation (4).

**Figure 3:**
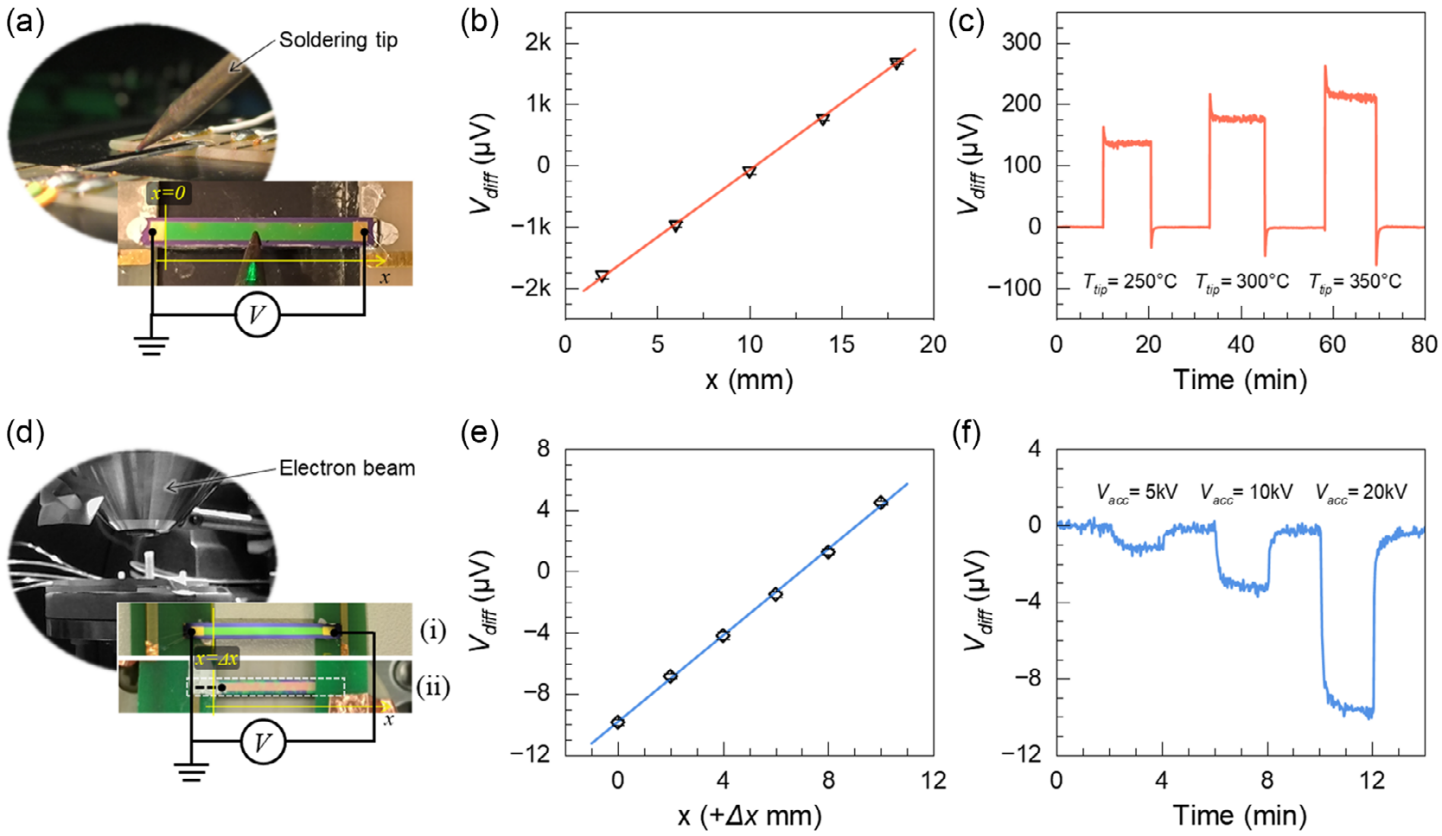
T‐PSD under multiple energy forms. a) Photographs of a 1D T‐PSD prototype working under a hot soldering tip as a HS. This device is 25 mm in length with a 20 mm active area. b) Measured *V*_diff_ at different positions along the detector using a hot soldering tip at 400 °C as a HS. c) The measured *V*_diff_ as a response to different soldering tip temperature at *x* ≈ 11.5 mm). The line is a linear fit with *R* ^2^ ≈ 1.0. d) Photographs of a 1D T‐PSD prototype working under an electron beam as a HS in a scanning electron microscope chamber. Top view (i) and bottom view (ii) of the prototype. To prevent incident electrons from affecting the detected signal, the back side of the detector is used to detect the electron beam. Besides, the rear surface has been coated with a 2 nm Au layer and is grounded with silver glue to prevent the impact of charge accumulation. This device is 20 mm in length with a 15 mm active area. e) Measured *V*_diff_ at different positions along the detector. Since the position *x* = 0 cannot be determined accurately, the position of the electron beam, *x +* Δ*x*, means the distance Δ*x* between the initial electron beam position and position *x* = 0, plus the distance *x* between the initial electron beam position and the current electron beam position. The line is a linear fit with *R* ^2^ ≈ 1.0. f) The measured *V*_diff_ as a response to different electron acceleration voltages at *x* = 0 + Δ*x* mm.

The generality of our methodology is demonstrated by using a hot soldering iron tip (**Figure** **3**a–c) as a HS and an electron beam (Figure 3d,e) instead of a laser beam. A different substrate, i.e., glass, is used to fabricate another prototype (Figure S1.5, Supporting Information), verifying that the T‐PSD does not rely on a semiconducting substrate. These results also reveal a robust position‐dependent response behavior of the T‐PSDs.

### 2.3 Implementation of 2D T‐PSD

**Figure 4:**
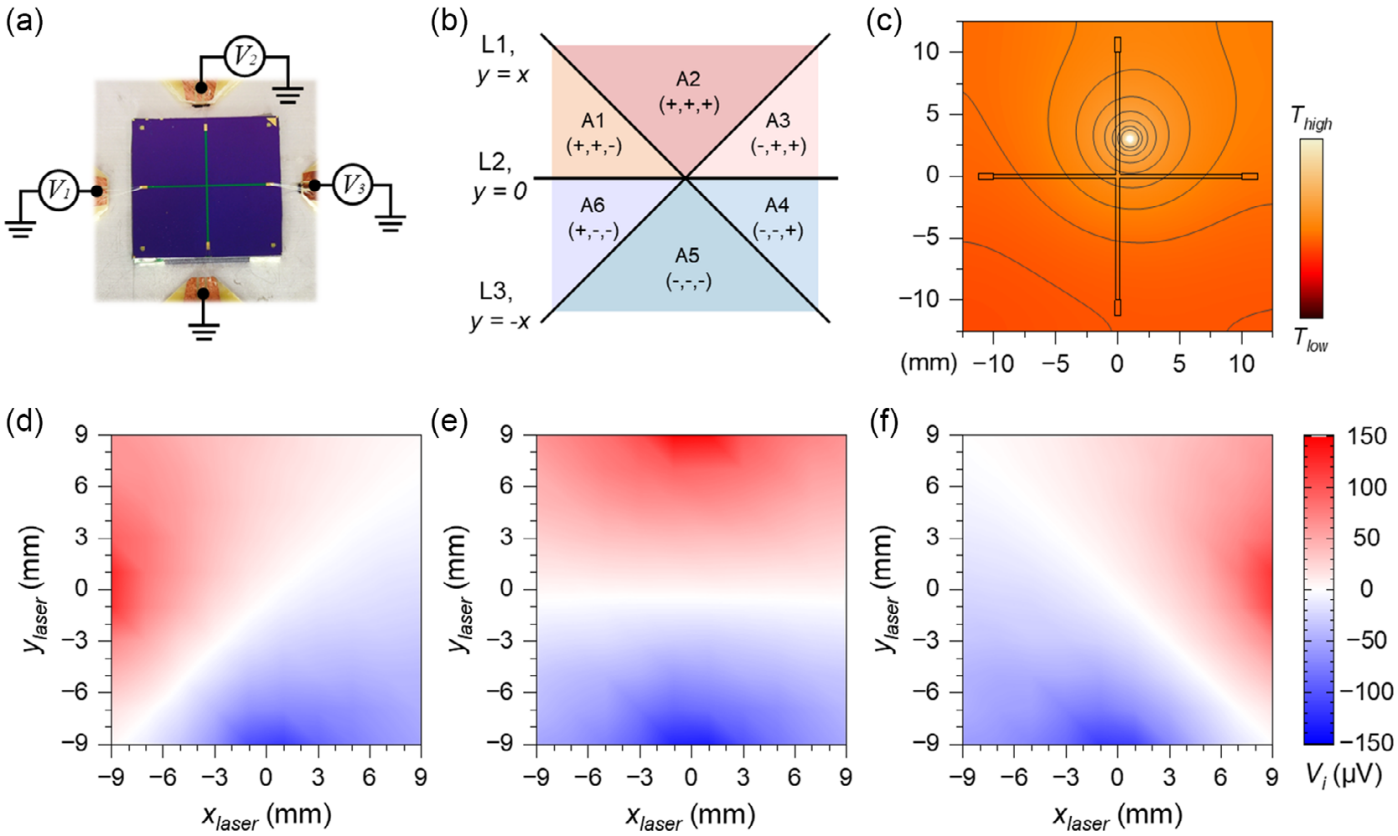
2D T‐PSD simulation and experimental results. a) Photograph of a 2D T‐PSD prototype, and corresponding circuit connection. b) The segmentation of the location area on the 2D T‐PSD for rough decoding. The position of the HS can be roughly judged by the sign combination of *V*_*i*_. c) The simulated temperature distribution on the substrate surface when a laser beam as HS is induced at the position (+1, +3). The prototype and the simulated model share the same size. d–f) The measured voltage signals *V*_*i*_ from the three voltage terminals, *V*_1_, *V*_2_, and *V*_3_ (as denoted in a), when the laser spot with PWM = 6% is located at different positions on the detector.

The 1D T‐PSD can be expanded into a 2D T‐PSD by changing the geometry of the thermoelectric thin film from a strip to a cross and integrating two additional voltage‐probe terminals, as shown in **Figure** **4**a and Figure S2.1., Supporting Information A mathematical model for 2D T‐PSD is derived by extending Fourier's law of heat conduction (Note 2.1). The following set of equations can be derived to express the probed thermoelectric voltage signals at the terminals

(5) $$\{\begin{array}{c} V_{1}=-\frac{qS}{4\pi kd}\text{ln}\left(\frac{{\left(x+L/2\right)}^{2}+y^{2}}{x^{2}+{\left(y+L/2\right)}^{2}}\right)\\ V_{2}=-\frac{qS}{4\pi kd}\text{ln}\left(\frac{x^{2}+{\left(y-L/2\right)}^{2}}{x^{2}+{\left(y+L/2\right)}^{2}}\right)\\ V_{3}=-\frac{qS}{4\pi kd}\text{ln}\left(\frac{{\left(x-L/2\right)}^{2}+y^{2}}{x^{2}+{\left(y+L/2\right)}^{2}}\right) \end{array}$$

where *V*_1_, *V*_2_, and *V*_3_ represent the detected signals at the different electrodes. *L* is the side length of the square substrate. The corresponding position of the HS is denoted by (*x*,*y*). The magnitude of the probed voltage signals is determined by the coefficient, $-\frac{qS}{4\pi kd}$, in Equation (5), which depends on the material properties and detector size, namely, *S*, *k*, and *d* as well as the heat flux *q* generated from the HS. According to the equation group, three special lines, where *V*_1_, *V*_2_, and *V*_3_ are equal to zero, can be highlighted. In detail, when the HS is positioned on the x‐axis, i.e., *y* = 0, *V*_2_ = 0. Also, when the HS is located on the diagonal, i.e., *y* = *x* or *y* = −*x*, the measured signal is *V*_1_ = 0 or *V*_3_ = 0, respectively. If any of the three signals equals zero, it indicates that the HS is located on the diagonals or *y* = 0 line. Additionally, the center of the 2D T‐PSD serves as a unique point because all signals are equal to zero. This feature can be used for precise centering scenarios. Furthermore, these three lines divide the detection area into six sections as displayed in Figure 4b. The combination of the positive and negative signs of the measured voltage signals *V*_1_, *V*_2_, and *V*_3_, as listed in Table S1, Supporting Information, can be used to roughly decode the position of the HS. Besides, the signal distribution within the x‐y coordinate system can be analyzed in a predictive manner, as depicted in Figure S2.2, Supporting Information.

Similar to the 1D case, we developed a FEA model to validate the voltage distribution patterns of the 2D T‐PSD design (Figure S2.3, Supporting Information). A spatial temperature distribution is observed when the sensor region is exposed to a HS, as seen in Figure 4c. Depending on the HS position, the temperatures of the different electrode terminals vary. The simulated *V*_1_, *V*_2_, and *V*_3_ distributions (Figure S2.4 and Figure S2.5a, Supporting Information) align well with the mathematical plots (Figure S2.2, Supporting Information). Afterward, we manufactured and assembled 2D T‐PSD prototypes (Figure 4a and Figure S2.6, Supporting Information) with a detection area of 18 × 18 mm^2^. When a CO_2_ laser beam, serving as a HS, is directed over the sensor's surface, it induces a localized temperature increase. This rise in temperature generates corresponding voltage signals at the different electrodes. A comprehensive comparison was conducted, illustrating the signal distributions from numerical calculations (Figure S2.2, Supporting Information), FEA simulation (Figure S2.4, Supporting Information), and experimental result (Figure 4d–f and Figure S2.5b, Supporting Information) for *V*_1_, *V*_2_, and *V*_3_. The distributions of both experimental and FEA results exhibit slight asymmetry and irregularity, primarily due to the influence of sensor geometry. Quantitative analysis reveals that the center of the experimental signal distribution is at (0.302, ‐0.453), which differs slightly from the numerical calculation and simulated signal centers, both located at (0, 0). Given the well‐controlled testing environment, we attribute this deviation mainly to imperfections of the sample itself, such as geometric irregularities from manual cutting, bonding connection defects, and contamination during the sample preparation process. A hot soldering iron tip is also used as a HS (Figure S2.7, Supporting Information) to prove the versatility of the T‐PSD in detecting diverse kinds of HSs, including but not limited to, laser beams, electrons, ions, to name a few.

To ensure the robustness and accuracy of the T‐PSD in practical scenarios, it is crucial to consider and mitigate the effects of various real‐world factors. Firstly, external environmental influences, such as ambient temperature fluctuations caused by convection, radiation exposure, uneven illumination, or nearby heat sources, can alter temperature gradients and lead to variations in the measured *V*_diff_. Besides, electromagnetic fields can also interfere with electron transport in the AZO film, causing noise and signal distortion. Such external factors can compromise the accuracy of the voltage signals. To mitigate these influences, experiments were conducted in controlled environments with constant temperature and electromagnetic shielding (Figure S2.8, Supporting Information). Secondly, experimental measurements often introduce noise and signal instability from electronic components, power supply fluctuations, or environmental electromagnetic interference, which can degrade voltage signal accuracy and lead to incorrect HS position detection. For this reason, it is necessary to collect the reference signal in advance to perform the necessary drift compensation. Our drift compensation strategy is illustrated in Figure S2.9, Supporting Information.

### 2.4 Decoding Pathways for 2D T‐PSD

In addition to the above control of the external environment and calibration during the measurement process, we developed two tailored decoding strategies for 2D T‐PSD. The decoding process can be generally categorized into two levels: rough decoding and accurate decoding. The varied sign combination of *V*_1_, *V*_2_, and *V*_3_ can be used to locate the HS (Figure 4b). For example, if the signs of *V*_1_, *V*_2_, and *V*_3_ are −, −, and +, respectively, indicating that the HS is situated in the A4 sector. One cannot derive the specific coordinate from this estimation except the center point. However, accurate decoding can be achieved through the “ratio strategy” we developed. The coefficient, $-\frac{qS}{4\pi kd}$, in Equation (5) affects the signal's amplitude, whereas the natural logarithm component governs the signal distribution and is only associated with the position (*x*, *y*). The ratios, for example, $\frac{V_{1}}{V_{2}}$, enable the elimination of the coefficients, making them independent of material properties and the local temperature rise due to the HS. Here, the following two ratios, *R*_a_ and *R*_b_, are selected for accurate decoding since both contain information about all three voltages

(6) $$\{\begin{array}{c} R_{a}=\frac{V_{1}}{V_{2}+\left(V_{1}-V_{3}\right)}\\ R_{b}=\frac{V_{3}}{V_{2}-\left(V_{1}-V_{3}\right)} \end{array}$$

Note, other ratios and combinations might be used as well if they lead to distinct solutions for the position (*x,y*). Practically, the accuracy of HS position detection can be compromised by signal inconsistencies due to variations in sensor performance, sensor shape, or external disturbances. Therefore, each T‐PSD should undergo complete offset calibration using reference HS positions to correct for systematic biases. This calibration ensures long‐term stability and accurate performance. The calibration procedure involves probing the entire detector surface first to obtain the line segment with the measured value *V*_*i*_ = 0, (*i = *1,2,3). Then, the measured positions are compared to the corresponding line segment with the theoretical value *V*_*i,*theoretical_ = 0 to obtain the offset data *x*_offset_ and *y*_offset_. For instance, according to the analysis in Figure S2.5, Supporting Information, the offset values are calibrated as., *x*_offset_ = 0.302 mm, *y*_offset_ = −0.453 mm for 2D T‐PSD in Figure 4. Then, the following formulas are used to correct the actual *x* and *y* in *V*_1_, *V*_2_, and *V*_3_ in Equation (5) and Equation (6),

(7) $$\{\begin{array}{c} x_{i,\text{update}}=x+x_{\text{offset}},\left(i=1,2,3\right)\\ y_{i,\text{update}}=y+y_{\text{offset}},\left(i=1,2,3\right) \end{array}$$

Note that the equation group can contain multiple solutions, i.e., several coordinates. During actual decoding, an important criterion for judging the correctness of the decoding result and the data quality is the proximity of these coordinate positions. We can anticipate a better decoding result the smaller the area of the coordinate groups present. From the coordinate set solved by *V*_1_, *V*_2_, and *V*_3_, take the average of all the solutions in the intersection set as the true solution. Then, calculate the mean and standard error of *x* and *y*, respectively, to get the coordinates $\left(\bar{x}\pm s_{x},\bar{y}\pm s_{y}\right)$. These coordinates represent the actual location of the HS.

**Figure 5:**
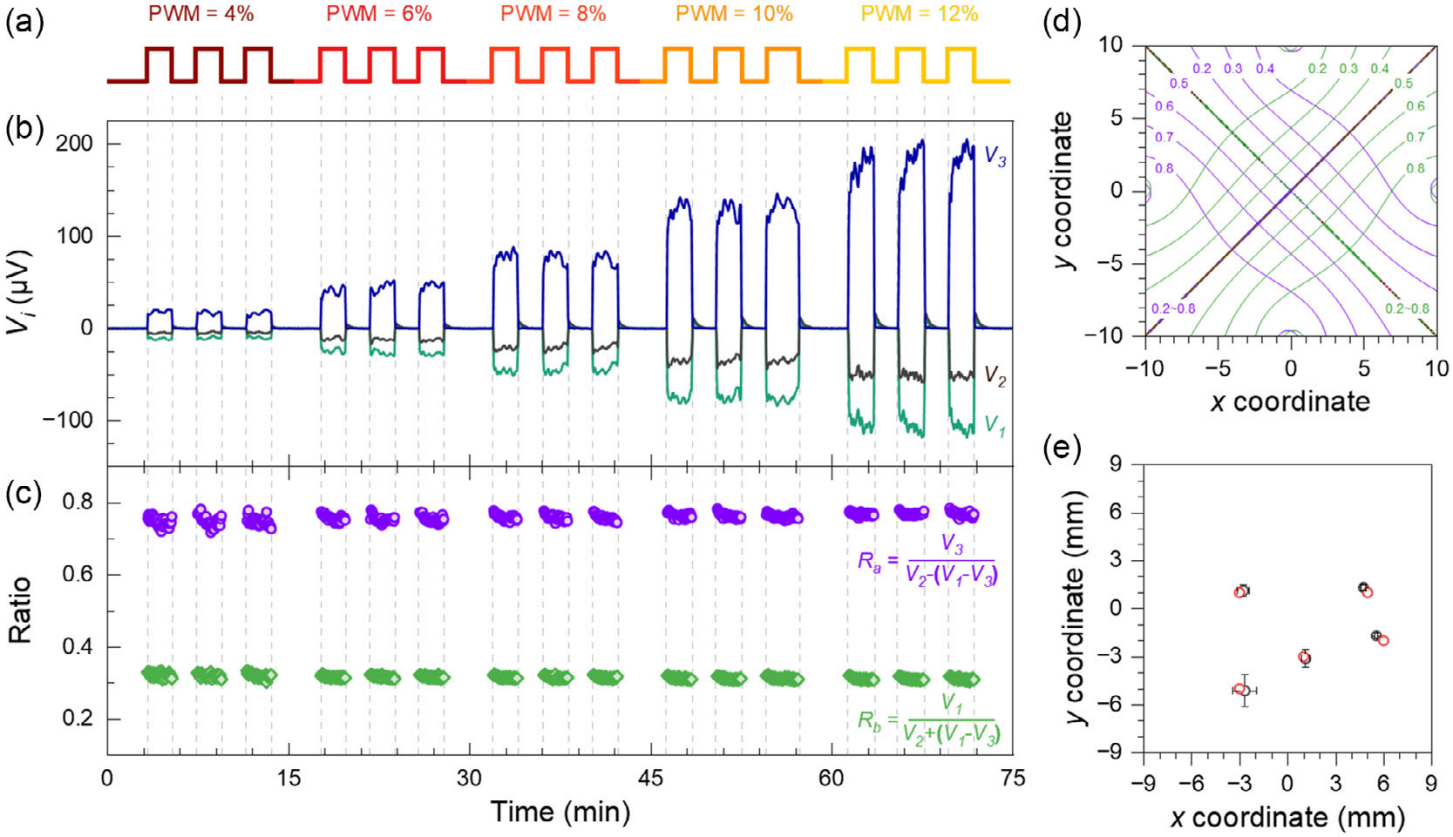
Demonstration of a method for accurate decoding the *V*_*i*_ into corresponding positions in 2D T‐PSD. a) Laser HS pulses setup. The laser spot is located at the position (6, −2) and varying PWM values of 4, 6, 8, 10, and 12% are used. b) The corresponding V_*i*_ signal. c) The derived ratios of *R*_a_ and *R*_b_. The ratio values remain almost unchanged despite changing the PWM values, suggesting their only position‐dependent character. d) Contour map of the calculated ratio values, *R*_a_ (green) and *R*_b_ (violet), over the detection area for accurate decoding. The precise position of the HS can be determined by comparison with this distribution. e) Comparison of several actual laser spot positions (red) with the corresponding decoded positions (black) using PWM = 6%.

These measures help to normalize the influence of signal amplitude variations, ensure stable measurements, and correct for systematic biases. By considering these factors, the T‐PSD can achieve reliable and precise heat spot location identification even under non‐ideal conditions. The reliability of the decoding is proven via a set of detected voltage signals at one position, i.e., (6, −2), under varying PWM values, as depicted in **Figure** **5**a,b. As predicted, the two ratios are independent of the characteristics of the HS, converting a fluctuating signal output into stable ratios for accurate decoding (Figure 5c and S3.1, Supporting Information). Each position in the detection area corresponds to a unique ratio pair, indicating that precise position decoding is feasible, as it is obvious in the contour diagrams of the two ratios (Figure 5d and S3.2, Supporting Information). To verify the reliability of the derived ratio strategy, several positions were randomly selected on the surface of the detector. The CO_2_ laser beam was used as the HS for this experiment. The actual positions ((−3, −5), (−3, 1), (1, −3), (5, 1), (6, −2)) and the decoded positions ((−2.66 ± 0.36, −5.13 ± 0.99), (−2.75 ± 0.36, 1.12 ± 0.38), (1.13 ± 0.28, −3.16 ± 0.53), (4.75 ± 0.15, 1.31 ± 0.18), (5.55 ± 0.11, −1.7 ± 0.12)) correspond well to each other, as shown in Figure 5e. Factors such as the detector's geometry, the air's convection, and the thermal radiation will be relatively weakened, when the temperature is higher. As predicted, the decoding is more accurate when the temperature rise induced by the laser's power is higher (Figure S3.3, Supporting Information). The results of the conducted experiments demonstrate the reliability, consistency, and stability in detecting a HS position using our T‐PSD concept.

## 3 Conclusion

In this study, we have introduced a new detector concept, termed the T‐PSD, designed for the precise position detection of a single HS through the analysis of generated thermoelectric voltages. Correspondingly, a reliable and precise decoding method, the “ratio strategy”, was proposed and demonstrated for extracting the spatial position information from the measured voltage signals. The fabricated prototypes have exhibited the capability to accurately detect HSs generated by various heat sources such as a laser beam, an electron beam, and a soldering iron tip. The comprehensive results highlight the T‐PSD's outstanding features, including high precision, exceptional sensitivity, robust repeatability, and positional stability. These characteristics have also been validated through analytical descriptions and FEA simulations. Nevertheless, potential enhancements for the T‐PSD encompass but are not limited to: (1) employing thermoelectric thin films with higher Seebeck coefficients; (2) considering the effects of heat convection, radiation, geometry effect of the substrate, and potential variations in the *S* and *k* with temperature; (3) incorporating additional electrode configurations for temperature reference or calibration. Throughout history, the introduction of new sensors has often facilitated advancements in various fields. The T‐PSD concept, characterized by its simplicity and solid grounding in physical principles, holds the promise of positively impacting various industrial domains in the near future.

## 4 Experimental Section

4.1

4.1.1

##### AZO Film Deposition by ALD

The AZO films were deposited in a super‐cycle ALD approach in exposure mode on SiO_2_ (300 nm)/Si wafers (SIEGERT WAFER GmbH) using a Savannah 100 reactor (Cambridge Nanotech). The deposition was performed at 200 °C with 30 sccm nitrogen as the carrier gas and a working pressure of ≈1.5 Torr. The precursors, namely, deionized water (DIW), diethylzinc (DEZ, Strem Chemicals, Inc., USA), and trimethylaluminum (TMA, Strem Chemicals, Inc., USA), were used as the source of O, Zn, and Al, respectively. All precursors were contained in stainless‐steel bottles and held at room temperature. An ALD super‐cycle was set as [(DEZ‐DIW)_a_ + (TMA‐DIW)_b_ + (DEZ‐DIW)_c_]_*x*_, to synthesize uniformly doped AZO film with a ratio of DEZ:TMA =(a + c):b = 20:1. The film thickness was adjusted to about 30 nm by running *x* = 30 super‐cycles.

##### The T‐PSD Device Preparation

Standard lithography processing was used to define the patterns, i.e., strip or cross for 1D and 2D, respectively, on the ALD‐deposited AZO film. Next, diluted HCl solution (HCl: H_2_O = 2:100 v v^−1^) was used to etch the film, leaving the desired pattern on the substrate. Subsequently, after removing the photoresist by acetone, another standard lithography process was used to define the electrode contact patterns, followed by sputtering Cr (10 nm)/Au (100 nm) as contacts.

##### Characterization

Seebeck coefficient of the AZO film was measured using a Potential‐Seebeck microprobe (PSM II, PANCO GmbH). The experimental tests using a CO_2_ laser (48‐2SWM, SYNRAD), whose machining resolution is smaller than 1 μm, and a hot soldering iron tip, as HSs were performed in a laser micromachining system (MM200‐Flex, OPTEC) within a metal protective cover. The power output is 25 W with a wavelength of 10.2–10.8 μm. The tests using an electron beam as HS were performed within the scanning electron microscope (Quanta FEG‐250). The average thermoelectric voltage signal from the PSD is measured using digital multimeters (34401 A, HEWLETT PACKARD HP) with an integration time of 1 s.

##### FEA Simulation

FEA conducted by COMSOL Multiphysics software was adapted to study the temperature and potential distribution on the T‐PSD. The three‐dimensional model was established using an one‐to‐one configuration (same size, same geometry) of the real T‐PSDs. A Gaussian beam profile was represented by the equation for the irradiance (intensity) distribution, $I\left(r\right)=\frac{P_{\text{laser}}}{2\pi r_{\text{spot}}^{2}}\text{exp}\left(-\frac{r_{\text{focus}}^{2}}{2r_{\text{spot}}^{2}}\right)$, where *P*_laser_ is the laser power, *r*_spot_ is the spot radius, and *r*_focus_ is the radius of the laser at the focus point. The experimentally measured Seebeck coefficient of −72.1 μV K^−1^ was assigned to the AZO film. In contrast to analytical description, a surface‐to‐ambient radiation was set to 0.8.

## Conflict of Interest

The authors declare no conflict of interest.

## Author Contributions

J.P. designed the sensor and experiments, fabricated the samples, and did the FEA analyses; J.P., P.Z., and S.H. set up the measurement system; J.P., R.V., and K.D. performed the measurements; R.Z. supervised the study; R.B. provided infrastructure to conduct the experiments. All authors analyzed data; J.P., R.B., and R.Z. wrote the manuscript. All authors have approved the final version of the manuscript.

## Supporting information

Supplementary Material

## Data Availability

The data that support the findings of this study are available from the corresponding author upon reasonable request.
