# Supplementary material for: Thermal Sight: A Position‐Sensitive Detector for a Pinpoint Heat Spot
Source: Small Sci. 2024 Jul 11;4(8):2400091. doi: 10.1002/smsc.202400091 (PMC11935274; doi:10.1002/smsc.202400091)
Supplement: Supplementary file 1 — Supplementary Material [file SMSC-4-2400091-s001.pdf]

Supporting Information

Thermal Sight: A Position-Sensitive Detector for a Pinpoint Heat Spot

Jun Peng, Pai Zhao, Rakshith Venugopal, Kristian Deneke, Stefanie Haugg, Robert Blick, Robert Zierold**

Content

Note 1. 1D T-PSD 2
Note 1.1. 1D T-PSD theoretical framework 2
Note 1.2. 1D T-PSD simulation 3
Note 1.3. 1D T-PSD experiments 4
Note 2. 2D T-PSD 6
Note 2.1. 2D T-PSD theoretical framework 6
Note 2.2. 2D T-PSD simulation 9
Note 2.3. 2D T-PSD experimental realization 11
Note 3. 2D T-PSD signals decoding 14

Note 1. 1D T-PSD

Note 1.1. 1D T-PSD theoretical framework

When a HS, e.g., a laser beam or a soldering iron tip, is present on the sensor surface, the heat transfer within the sensor can be described by Fourier's law of heat conduction. This fundamental law states that the rate of heat transfer through a solid is proportional to the temperature gradient, and can be mathematically expressed as

$$q = -kA_{1D}\frac{dT}{dx} = -kwd\frac{dT}{dx}, \qquad \text{(S1-1)}$$

where q is the heat flux density, k is the material's thermal conductivity, and is considered a constant here, A_{1D} is the area for the heat flow. To simplify the calculation, we neglected the thickness of the thermoelectric film as it is very thin (<100 nm). So, the A_{1D} is the cross-section area of the substrate along the x-axis, i.e., $A_{1D} = wd$, where w and d are the width and thickness of the substrate, respectively. dT/dx is the temperature gradient, i.e., the change in temperature with respect to the distance in the direction of the heat flow. We also neglected the convection and radiation between the environment and the sensor, and only consider the lateral heat transfer within the substrate. This equation shows that the temperature distribution in a 1D system with a constant HS is linearly proportional to the distance from the HS. Combining equation (S1-1) and the Seebeck effect expression, equation (2), we have

$$\frac{dV}{dx} = \frac{qS}{kA_{1D}}. \qquad \text{(S1-2)}$$

Assuming a steady-state condition, where a HS with a constant temperature T_0 is located at $x = x_0$, the temperature distribution does not change with time. Two defined temperatures, T_1 and T_2, occur at the two ends of the sensor, i.e., at points $x(P_1) = -L/2$ and $x(P_2) = L/2$, respectively. The thermoelectric voltage, V_{diff}, generated by the temperature difference can be described as

$$V_{diff} = \frac{qS}{kA_{1D}}\left(\left(\frac{L}{2} + x_0\right) - \left(\frac{L}{2} - x_0\right)\right) = \frac{qS}{kwd}(2x_0). \qquad \text{(S1-3)}$$

Consequently, V_{diff} reflects the position of the HS. This potential can be probed easily by a multimeter connected between the terminals.

Note 1.2. 1D T-PSD simulation

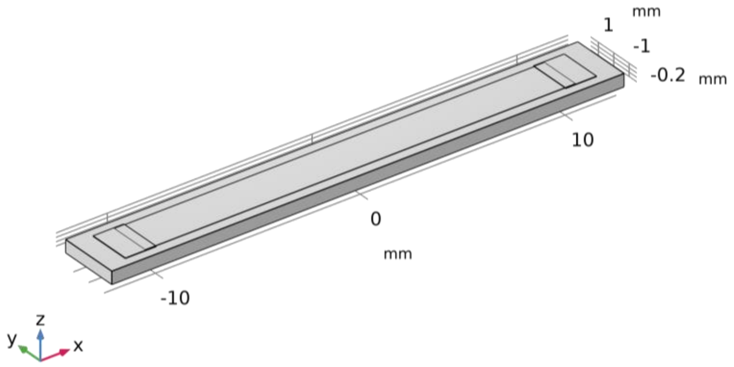

Figure S1.1. The simulation model geometry for the 1D T-PSD.

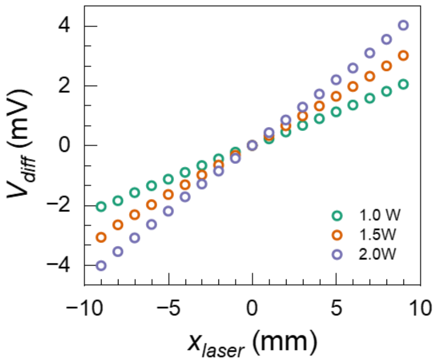

Figure S1.2. Simulated V_{diff} at different laser powers on the 1D T-PSD. They all reveal a linear relationship with the x-coordinate.

Note 1.3. 1D T-PSD experiments

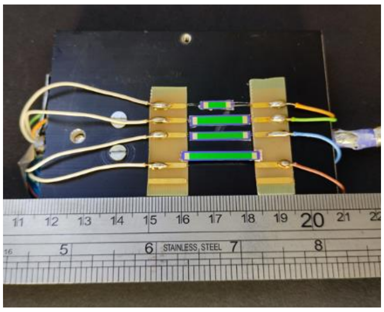

Figure S1.3. Photograph of several 1D T-PSDs on the test holder. Note that the longest T-PSD is elevated on the printed circuit board to prevent the holder's influence.

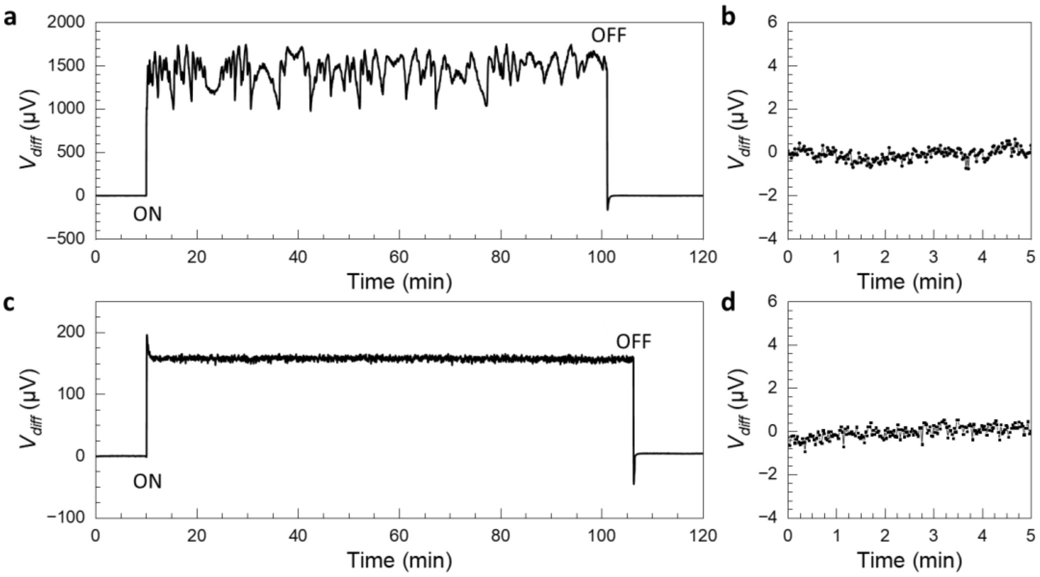

Figure S1.4. Comparison of the V_{diff} using different HS. (a) Overview of the probed V_{diff} from a long-time measurement using a CO_2 laser beam with PWM=6% as a HS. (b) Zoomed-in base signal in (a). (c) Overview of the probed V_{diff} from a long-time measurement using a hot soldering iron tip with the temperature set to 300 °C as a HS. (d) Zoomed-in base signal in (c). The small fluctuation in (b) and (d) is the noise from the multimeter, while the stronger fluctuation in (a) when the laser is ON comes from the unstable laser output. The applied CO_2 laser is a cutting laser with a power stability of ±5% without a voltage regulator.

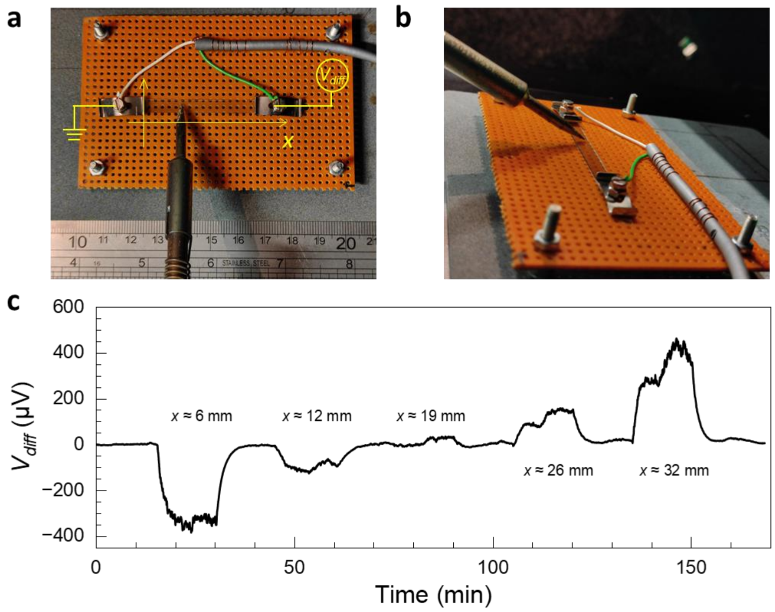

Figure S1.5. Glass substrate with an AZO film on top for a 1D T-PSD. (a) Top view and (b) bottom view of the glass-based 1D T-PSD device using a hot soldering iron tip as a HS. This device is 50 mm in length with a 38 mm active area. (c) The measured V_{diff} when the soldering iron tip with a temperature of 400 °C is located at different positions.

Note 2. 2D T-PSD

Note 2.1. 2D T-PSD theoretical framework

Expanding the substrate of the 1D T-PSD and distancing the HS away from the thin thermoelectric film strip will lead to diminished accuracy in position detection. This reduction in sensitivity occurs because a variation in the distance between the electrodes at both ends is more sensitive to movements of the HS along the linear axis defined by the two probe electrodes, as opposed to movements perpendicular to this axis. To address this limitation, a cross-shaped thermoelectric film is introduced, transforming the 1D T-PSD into a 2D T-PSD. We still ignore the thickness of the thermoelectric film and the convection between the environment and the sensor as well as its own thermal radiation. Only the lateral heat transfer is considered within the substrate. Furthermore, we assume the 2D sensor as an infinite 2D plate. Within this 2D space, the temperature distribution, as shown in Figure S2.1a, is a function only of radial distance r between the point heat source (x, y) and the probe points (x_i, y_i), with $i = 1, 2, 3$, and is independent of azimuth angle when using the polar coordinates, which can be expressed as

$$r = \sqrt{(x - x_i)^2 + (y - y_i)^2}. \tag{S2-1}$$

Again, Fourier's law is used by inserting the proper area relation A_r, taking the substrate thickness d into account. The heat transfer can be considered as a radial transfer mode within a 2D plate. The area for the heat flow can be written as $A_r = 2\pi rd$. The radial heat flux q_r from the HS is then written as

$$q_r = -kA_r \frac{\partial T}{\partial r} = -2\pi krd \frac{\partial T}{\partial r}, \tag{S2-2}$$

with the boundary conditions $T = T_i$ at $r = r_i$, and the HS temperature $T = T_0$ at the HS edge $r = r_0$. The solution to the above equation is

$$q = -2\pi kd \frac{(T_i - T_0)}{ln(r_0/r)}. \tag{S2-3}$$

Then temperature $T(x, y)$ at any point (x, y) on the surface with a HS at position (x_0, y_0) and a constant temperature T_0 can be expressed using the below equation

$$T(x, y) = T_0 - \frac{q}{2\pi kd}\ ln(\frac{r_0}{r_i}), \tag{S2-4}$$

Where r_0 is the radius of the HS. According to equation (S2-4), the temperature distribution is relatively insensitive to small variations in the value of r_0. The T-PSD detects the position but not the shape of the HS.

As shown in Figure S2.1b, the 2D T-PSD has one ground and three probe terminals at $P_1(-L/2,0)$, $P_2(0,L/2)$, and $P_3(L/2,0)$, respectively. The temperature difference between the three points and the ground point $P_4(0,-L/2)$ can be written as

$$\begin{cases} \Delta T_{1-4} = T_1 - T_4 = T(-L/2,0) - T(0,-L/2) = \frac{q}{2\pi kd}\ ln(\frac{r_1}{r_4}) \\ \Delta T_{2-4} = T_2 - T_4 = T(0,L/2) - T(0,-L/2) = \frac{q}{2\pi kd}\ ln(\frac{r_2}{r_4}) \\ \Delta T_{3-4} = T_3 - T_4 = T(L/2,0) - T(0,-L/2) = \frac{q}{2\pi kd}\ ln(\frac{r_3}{r_4}) \end{cases}. \quad \text{(S2-5)}$$

Combining equation (S2-1) and equation (S2-5), we have

$$\begin{cases} V_1 = -S\Delta T_{1-4} = -\frac{qS}{2\pi kd}\ ln\left(\frac{r_1}{r_4}\right) = -\frac{qS}{4\pi kd}\ ln\left(\frac{(x+L/2)^2+(y)^2}{(x)^2+(y+L/2)^2}\right) \\ V_2 = -S\Delta T_{2-4} = -\frac{qS}{2\pi kd}\ ln\left(\frac{r_2}{r_4}\right) = -\frac{qS}{4\pi kd}\ ln\left(\frac{(x)^2+(y-L/2)^2}{(x)^2+(y+L/2)^2}\right) \\ V_3 = -S\Delta T_{3-4} = -\frac{qS}{2\pi kd}\ ln\left(\frac{r_3}{r_4}\right) = -\frac{qS}{4\pi kd}\ ln\left(\frac{(x-L/2)^2+(y)^2}{(x)^2+(y+L/2)^2}\right) \end{cases}. \quad \text{(S2-6)}$$

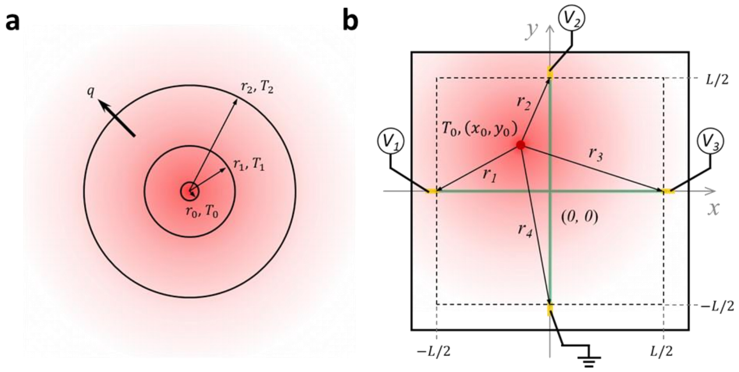

Figure S2.1. Heat conduction sketch in 2D space. (a) Polar coordinates. (b) The calculation in 2D T-PSD.

When the three thermoelectric voltages, V_1, V_2 and V_3, are measured, the above equation group can be utilized to derive the HS position (x, y) and the heat flow q. In equation (S2-6), the coefficient, $-\frac{qS}{2\pi kd}$, remains independent of the position and is solely related to the sensor material. Hence, the coefficient can be normalized to a constant, e.g., 1, for studies focusing solely on position dependence. By plotting the mathematical model with a normalized coefficient, as illustrated in Figure S2.2, the resulting patterns of the graphs closely align with the experimental data displayed in Figure 4d-f. These agreements provide compelling evidence that our proposed fitting model is accurate.

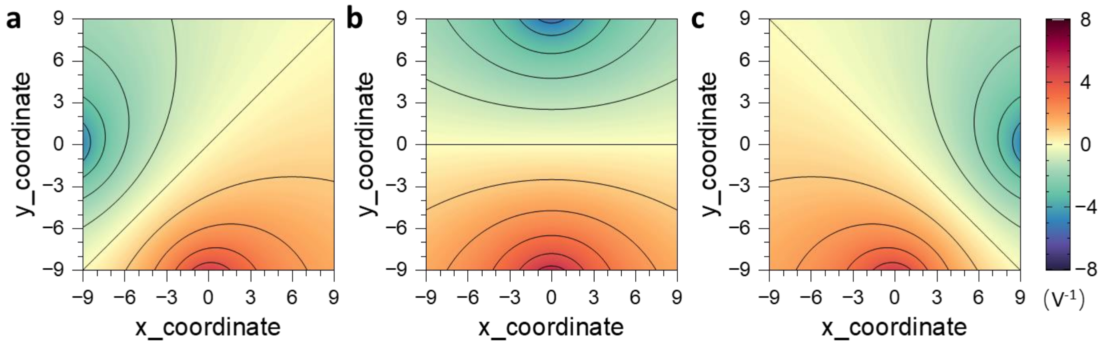

Figure S2.2. The analytical calculated V_i signal. They are the function graphs pictured from the mathematical model for (a) V_1, (b) V_2, and (c) V_3, respectively, in equation (S3-6).

Note 2.2. 2D T-PSD simulation

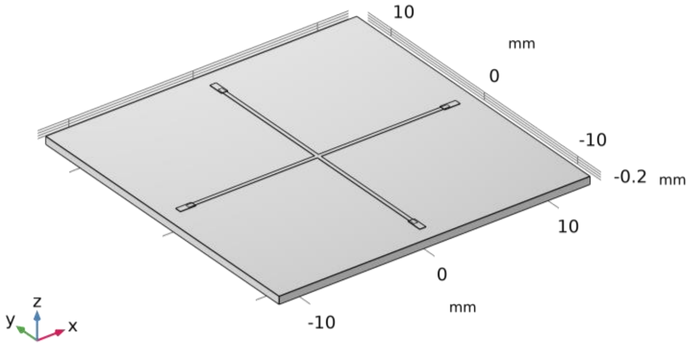

Figure S2.3. The simulation model geometry for 2D T-PSD.

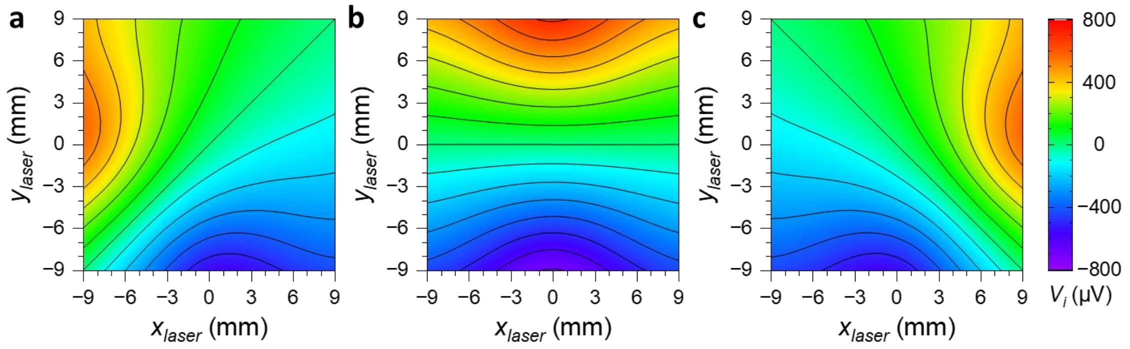

Figure S2.4. The simulated V_i signal. Simulated voltages, V_1, V_2, and V_3, (panel (a), (b), and (c), respectively) when the laser is located at different points on the 2D T-PSD surface. The laser power used for the simulation is 2W.

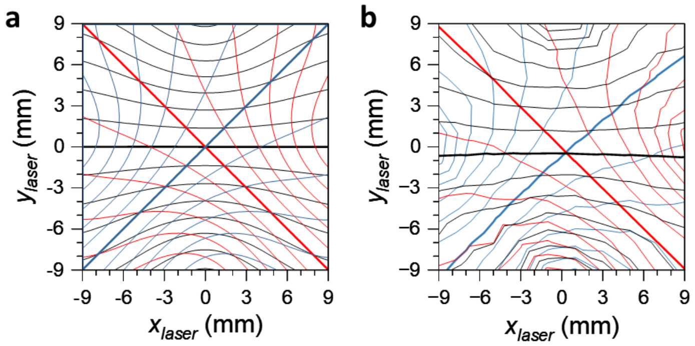

Figure S2.5. Contour plot comparison of simulated and experimental data. (a) Contour plot integration of voltage distribution obtained from FEA simulation. (b) Integration of contour maps of experimental voltage distributions. In these plots, the blue, black, and red lines represent the corresponding distributions of V_1, V_2 and V_3 respectively. The thick blue, black, and red lines represent the corresponding distributions, where $V_1 = 0$, $V_2 = 0$ and $V_3 = 0$ respectively. In the simulated distribution, the center point, where $V_1 = V_2 = V_3 = 0$, is located at the exact center of T-PSD, at coordinates (0, 0). However, due to imperfect sample cutting, the distribution obtained from experimental testing does not exactly match the simulated distribution. The intersection points between the three lines in the experimental distribution are (0.302, -0.400), (0.223, -0.479), and (0.380, -0.479). The center point of these three intersections, (0.302, -0.453), is taken as the distribution center of the measured signal. These coordinates are used for data correction offsets, i.e., x_{offset} = 0.302 mm, y_{offset} = -0.453 mm.

Note 2.3. 2D T-PSD experimental realization

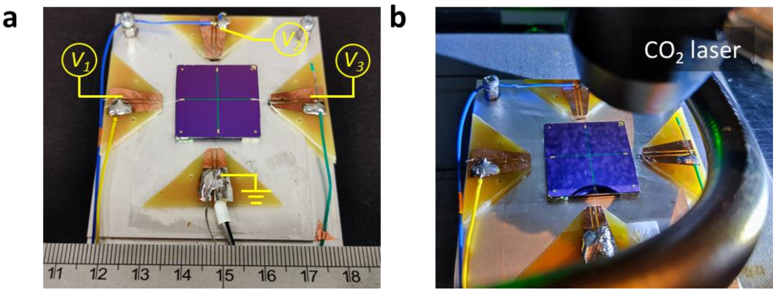

Figure S2.6. Photograph of a 2D T-PSD prototype. The 2D T-PSD is (a) integrated onto the test holder and (b) implemented in the test environment using a CO_2 laser beam as a HS.

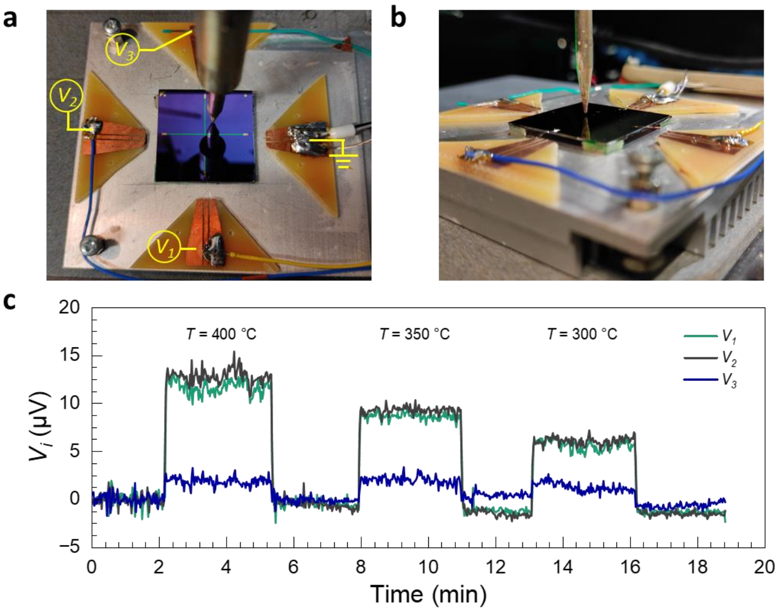

Figure S2.7. Hot soldering iron tip as a HS for 2D T-PSD. (a) Top view and (b) side view of the 2D T-PSD device. This device is the same 2D T-PSD device used in Figure S2.6. (c) The measured V_{diff} under different temperatures when the soldering iron tip is located near (-7, 7) mm.

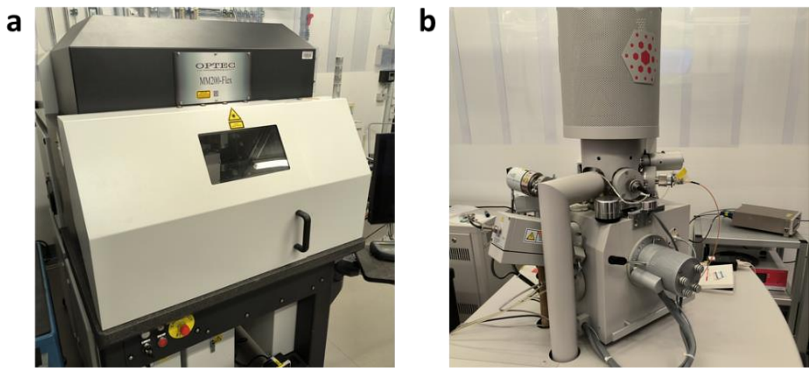

Figure S2.8. Protection of test environment. (a) All tests using the CO_2 laser beam and the hot soldering iron tip as HS were conducted within the metal protective cover of the laser micromachining system. (b) All tests using the electron beam as the heat source were performed in the high vacuum metal chamber of the scanning electron microscope. These tests were conducted in a laboratory with constant temperature and humidity to ensure the stability of the experimental environment.

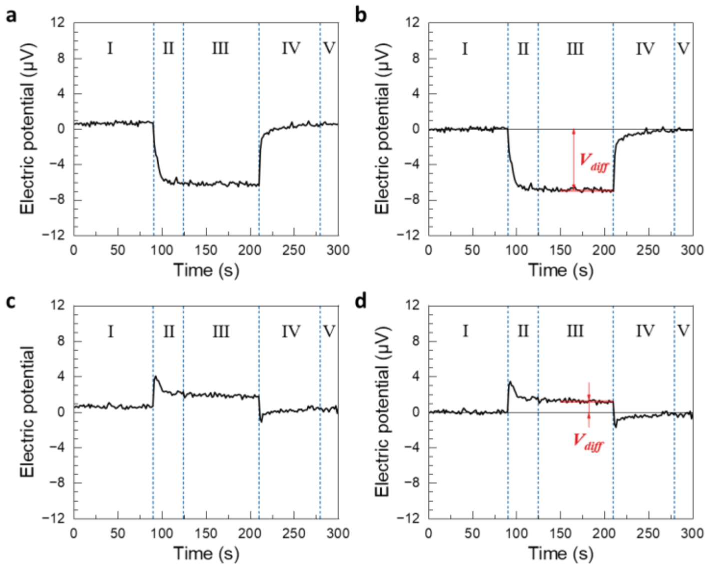

Figure S2.9. Typical signal analysis to show the drift compensation strategy. (a) and (c) are the signals when the electron beam is located at x=2+Δx mm and x=8+Δx mm, respectively. These are representative thermoelectric DC signals for stable HS measurements, with each test taking about 5 minutes. The HS is not on the detector for the first 1.5 minutes (Phase I), during which the temperature is uniform across the detector, and the measured signal during this phase serves as a reference baseline to correct for multimeter drift. Oscillations in the baseline are due to test noise. Upon activating the HS, the temperature gradient begins to form, changing the sample signal. However, it takes some time for the temperature gradient to

stabilize, so the measured signal also takes a period (Phase II) to relax and reach a stable state (Phase III). After removing the HS, the sensor's temperature gradually cools down (stage IV) and returns to room temperature (stage V). In order to improve the accuracy of decoding, the average value of the baseline is taken as the drift of the multimeter, and the overall signal is shifted and corrected based on this drift value. The drift-corrected signals for the electron beam at x=2+Δx mm and x=8+Δx mm are shown in (b) and (d), respectively. To ensure signal accuracy, the average value of the last segment of data in the stage III signal is taken as V_{diff} for position decoding. For instance, in these two examples, we select the average value of the data from one minute before removing the HS as V_{diff}.

Note 3. 2D T-PSD signals decoding

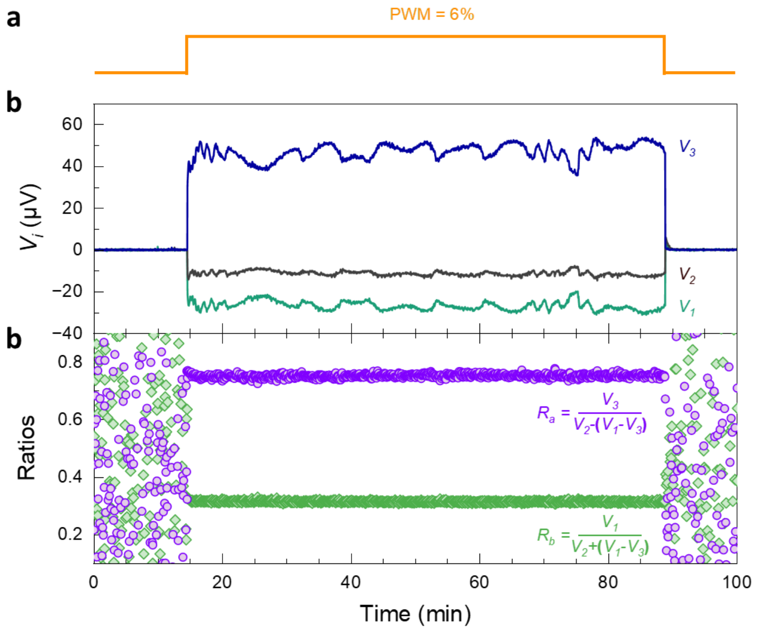

Figure S3.1. Long-time measurement for a 2D T-PSD at the position (6, -2) to highlight the stability of the ratios. (a) Laser pulse sketch. (b) The corresponding V_i signala. (c) The corresponding derived ratios of R_a and R_b. The ratio remains stable throughout the measurement. The position determination is independent of the form of the HS and can even attenuate the instability of the HS. These ratios are offset by the coefficient, $-\frac{qS}{2\pi kd}$, that allow them to be independent of material properties, the local temperature of the HS, and even its inherent fluctuations.

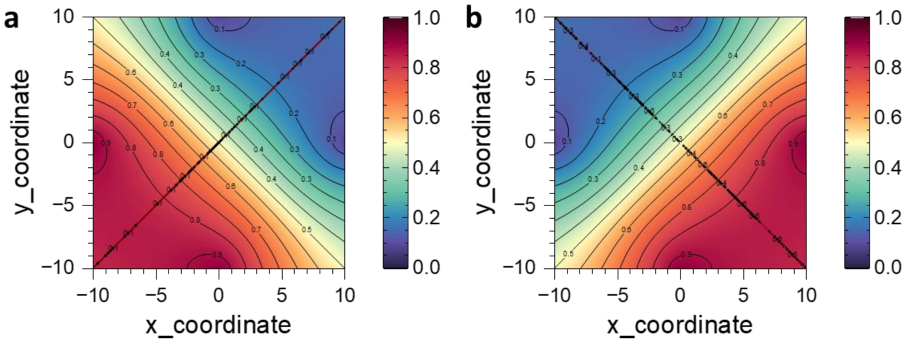

Figure S3.2. The graphs from the mathematical model for (a) R_a and (b) R_b.

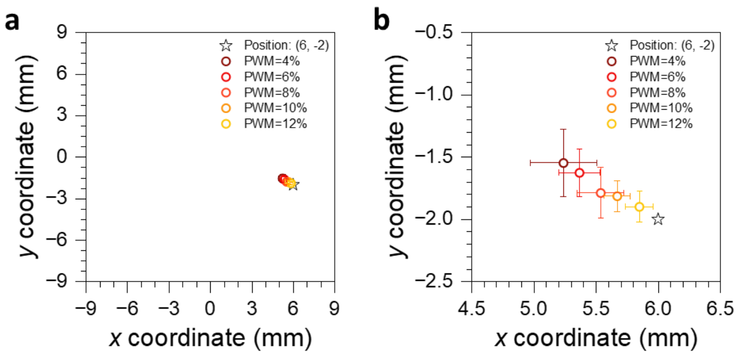

Figure S3.3. The decoded result comparison for different PWM values in Figure 4b. The signal of the second laser pulse under each PWM setting is used. within (a) the whole detecting area and (b) the zoomed-in area. Although position (6, -2) is already on the edge of the accurate area, a higher PWM will make the decoding more accurate due to the weakened environment influence.

Table S1. The sign combination of the measured signals.

Region/Line	V_1	V_2	V_3
L1	0		
L2		0	
L3			0
A1	+	+	-
A2	+	+	+
A3	-	+	+
A4	-	-	+
A5	-	-	-
A6	+	-	-
Center point	0	0	0
